# 

**Published:** 1868-08-01

**Authors:** 


### CONTENTS:

ORIGINAL PAPERS:

Academy of Sciences, Paris—Experimental Physiology. Translated by Walter Hay, M.D., Associate Editor ... 475
Syphilization as Treatment for Syphilis ... 488
Proceedings of the Chicago Medical Society ... 492
Remarkable Case of Placenta Prævia. By Hiram Wanzer, M.D., Chicago, Ill. ... 493
Biliary Calculi. By E. W. Boyles, M.D., Clay City, Ill. ... 497
Notes and Observations in General Practice. By John Forman, M.D., Edinburgh ... 499

CORRESPONDENCE ... 505

EDITORIAL:

The Western Tripod of Medicine ... 507
The Inconveniences of being a Good Fellow ... 510
Extra Eight Pages ... 511
Arrearages ... 511
Peccavimus ... 512
Chicago Hotel for Invalids ... 513

FOREIGN NOTES AND EXCERPTA ... 513

